# *TLR7* mutations leading to enhanced TLR7 signaling in humans

**DOI:** 10.70962/jhi.20250266

**Published:** 2026-02-24

**Authors:** Yanick J. Crow, Michael P. Gantier

**Affiliations:** 1 MRC Human Genetics Unit, Institute of Genetics and Cancer, University of Edinburgh, Edinburgh, UK; 2 Laboratory of Neurogenetics and Neuroinflammation, INSERM UMR 1163, Imagine Institute, Université Paris Cité, Paris, France; 3 Centre for Innate Immunity and Infectious Diseases, Hudson Institute of Medical Research, Clayton, Australia; 4 Department of Molecular and Translational Science, Monash University, Clayton, Australia

Systemic lupus erythematosus (SLE) encompasses a spectrum of autoimmune phenotypes, ranging from isolated cutaneous involvement to multi-organ systemic disease, characterized by the presence of autoantibodies targeting nuclear antigens and type I interferon upregulation (1). While most cases of lupus are considered to have a multifactorial basis (2), it has been suggested that 5–10% of early-onset SLE might result from highly penetrant single gene mutations (3).

The human Toll-like receptors (TLRs) constitute a family of 10 proteins engaged by microbial features as part of a coordinated innate and adaptive immune response to pathogens (4), with TLR3, TLR7, TLR8, and TLR9 trafficking from the endoplasmic reticulum to the endosomal compartment, where they sense and respond to specific nucleic acid species. Notably, TLR7 and TLR8 are encoded in close proximity on the X chromosome and share strong structural and functional homology. Both receptors are involved in sensing short RNA degradation products, which can be either pathogenic- or self-derived. Reflective of the latter situation, disease in murine models of SLE is attenuated in *Tlr7*-deficient animals, and TLR7 over-expression in mice can drive SLE-like pathology. In 2022, Brown et al. provided the first description of humans carrying heterozygous mutations in *TLR7* leading to enhanced TLR7 signaling, with disease variably manifesting as either SLE or aquaporin 4 autoantibody positive (AQP4^+^) neuromyelitis optica (NMO) in the absence of lupus (5). Additional case reports followed in 2024 (6, 7, 8) and 2025 (9), highlighting the high risk of both SLE and neuroinflammation. In this issue of the *Journal of Human Immunity*, three reports (10, 11, 12) further extend the mutational landscape and phenotypic spectrum associated with increased TLR7 activity due to mutations in *TLR7*. Notably, several structurally equivalent mutations in TLR8 have also been described as pathogenic, although they do not result in SLE phenotypes likely due to differences in the tropism of TLR8 expression (13).

Including the new papers published here, there are now eight reports in the literature describing 15 *TLR7* mutation–positive individuals from 11 families (Table 1). Genetically, all patients are heterozygous (or hemizygous in the case of affected males) for a variant leading to a missense substitution in TLR7. In seven probands the mutation arose de novo (with one case being a somatic mosaic); in three probands, the mutation was maternally inherited, and in one, inheritance was undetermined. Given that *TLR7* is X-linked, the observation of a preponderance of female, as opposed to male, mutation-positive individuals (12:3) is of possible note. Previously, the only hemizygous males described with such *TLR7* mutations both presented within the first few weeks of life, one with a neurological disorder reminiscent of severe Aicardi-Goutières (AGS) (6) and the other, mosaic for a F506S mutation, with very early onset SLE (9). The description by Sethumadhavan et al. (12) of a male with a germline L840R mutation, manifesting treatment-responsive lupus and no neurological disease at age 22 years, perhaps suggests that the mutation in this case is less deleterious from a molecular perspective.

Of the 14 mutation-positive individuals with clinical data (noting that in the paper by Sethumadhavan et al. the clinical status of the mother is unknown), disease onset was by age 2 years in seven patients, within the first decade of life in 11, and by age 18 years in 13, with a single individual presenting at 25 years of age. SLE and or autoimmune thrombocytopenia/anemia were seen in the majority of cases. From a neurological perspective, both antibody-mediated disease (anti-N-methyl-D-aspartate [NMDA] encephalitis; AQP4^+^ NMO) and features consistent with those seen in AGS and other type I interferonopathies were observed in 10 of 14 patients for whom clinical data were available. As comprehensively summarized by Tusseau et al. (11), overall, disease was severe and frequently refractory to standard lines of therapy, with three individuals undergoing hematopoietic stem cell transplantation (HSCT). While HSCT led to sustained remission of autoimmune and hematologic manifestations, the efficacy of HSCT in reversing neuroinflammation remains unclear at this time.

**Figure:**
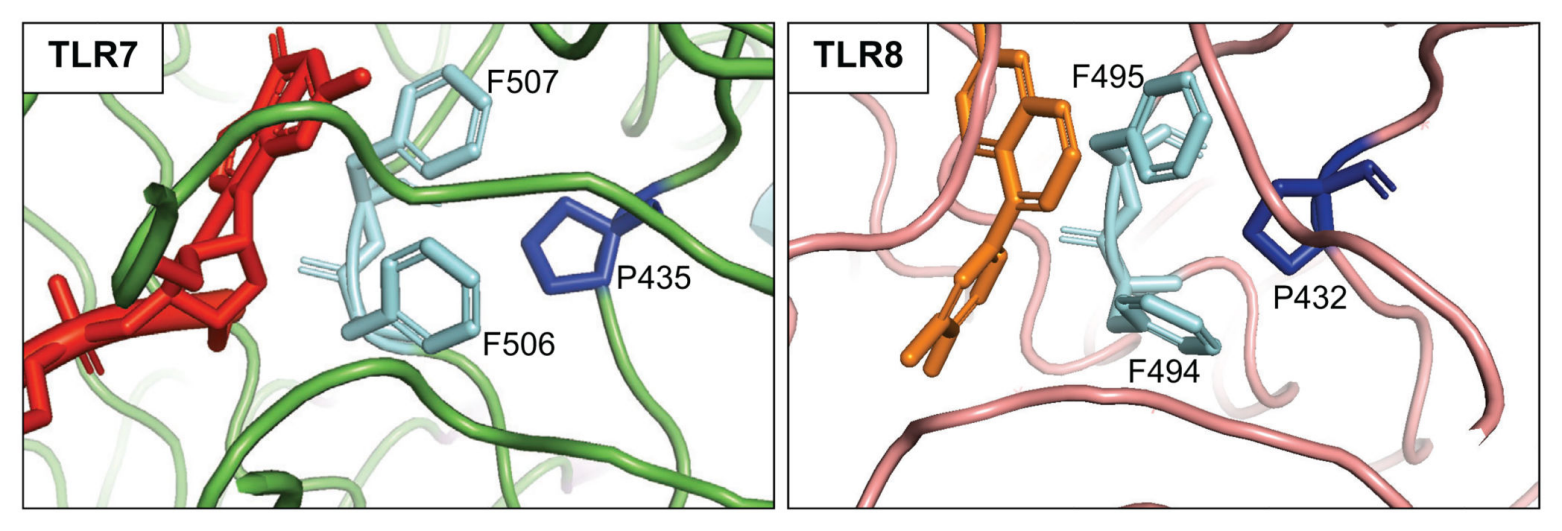
Structural representation of antagonistic-binding sites in TLR7 and TLR8. Structures of *Macaca mulatta* TLR7 (left—from PDB: 9LUV) and human TLR8 (right—from PDB: 5WYZ) antagonist-binding sites in complex with their respective antagonists. For TLR7, the antagonist shown in red is a three-base RNA with a 2′-O-methyl guanosine (mGrUrC), which faces the residues F506/F507 (shown in light blue). For TLR8, the small molecule CuCPT9b antagonist shown in orange faces the residues F494/F495 (shown in light blue). The molecular interactions between the antagonists and the F506/F507 and F494/F495 residues are essential for binding to TLR7 and TLR8, respectively. In addition, the P435 and P432 residues (shown in dark blue) indirectly contribute to the formation of the antagonistic-binding sites. Mutations of these specific residues, found in patients, contribute to aberrant inflammation by impairing natural antagonism of these receptors.

TLRs are type I transmembrane proteins with an extracellular or luminal Leucine rich repeatss (LRR) domain, a single transmembrane helix, and a cytoplasmic Toll/interleukin-1 receptor domain. Upon agonist binding, TLR dimers recruit downstream signaling adaptors eliciting inflammatory responses. TLR7 and TLR8 contain two distinct agonist-binding sites, recognizing single stranded RNA (ssRNAs) in the form of their degradation products, nucleosides, and oligoribonucleotides. Site 1, which is highly conserved between TLR7 and TLR8, recognizes both nucleosides (guanosine for TLR7, uridine for TLR8) and nucleoside analogues and is essential for receptor dimerization. In contrast, site 2 is less well conserved and is engaged by short uridine rich fragments (e.g., UUU and UG), enhancing the binding affinities of site 1 ligands.

Previous studies have demonstrated that sugar and base modifications of uridines, guanosines, and adenosines can attenuate the sensing of otherwise potent TLR7 and TLR8 agonistic RNAs, providing a plausible explanation for how endogenous RNAs harboring such modifications might not elicit an immune response. However, a growing body of evidence now indicates that TLR7 and TLR8 can detect very short RNA degradation fragments of 1–3 bases, a length at which the majority of endogenous RNA fragments are not modified. This latter finding raises the question of how such phagocytosed fragments of self-RNAs evade aberrant recognition in homeostasis. In this context, Alharbi et al. (14) now report that select 2′-O-methyl (2′-OMe) guanosine RNA fragments, including those derived from abundant host-ribosomal RNAs, are potent TLR7 and TLR8 antagonists that reduce TLR7 sensing in vivo.

Mechanistically, these antagonistic fragments are directed by 5′-end 2′-OMe guanosine toward a third binding site in TLR7 and TLR8, distinct from the previously described sites 1 and 2, thereby triggering the formation of an inactive open dimer. These data suggest a model where host RNAs do not trigger signaling by TLR7/8 due to the presence of a pool of abundant host ribosomal 2′-OMe-modified RNA fragments that basally antagonize TLR7 and TLR8 sensing. Crucially, substitutions in recombinant TLR7 at P435, F506, and F507 (residues mutated in five of the 11 families summarized here), and in recombinant TLR8 at F495 (structurally and functionally equivalent to the F507 residue in TLR7), decreased the binding affinity of inhibitory 2′-OMe guanosine RNAs in surface plasmon resonance (SPR) analyses (14). In addition to structural analyses describing how 2′-OMe guanosine RNAs bind to the F506/507 (TLR7) and F494/495 (TLR8) regions, these studies provide a definitive mechanistic understanding of how mutation of these residues alters antagonism mediated by 2′-OMe guanosine RNAs. While it remains possible that additional structural variations arising from these mutations might enhance site 1 activity to guanosine, in the experiments reported by Alharbi et al. there was no associated increase in binding of R848.

Collectively, the above new data argue that mutations at residues P435, F506, and F507 in TLR7 result in autoimmunity by compromising the antagonistic activity of a pool of inhibitory 2′-OMe guanosine RNA fragments that act to keep TLR7 and TLR8 inactive in steady state. Put another way, the findings of Alharbi et al. suggest that, despite enhanced TLR-mediated pro-inflammatory signaling, at least some mutations in TLR7 and TLR8 result in a loss of function at a molecular level. This point is of more than academic importance because specific TLR7/8 inhibitors, now in early clinical trials, might only be expected to work in the case of a molecular gain of function. Possibly instructive in this regard is a recent observation reported by Aluri et al. relating to mutations in TLR8 (13). In the majority of cases reported to date, disease results from somatic mutations involving a proline at position 432. Notably, P432 in TLR8 lies in the equivalent position to P435 in TLR7, essential to the binding of antagonistic 2′-OMe-modified RNA (see above). Of note then, while CU-CPT9a (a selective inhibitor of TLR8 signaling that binds to the antagonistic pocket of TLR8 preventing downstream signaling) was able to suppress the activity of stimulated wild-type TLR8, it failed to inhibit signaling in the context of the P432L mutant. Consistent with the data relating to P435, F506, and F507 in TLR7 described by Alharbi et al., this result supports the possibility that mutations in TLR8 involving P432 also act through a loss of the antagonistic potential of endogenous RNAs, rather than a molecular gain of function.

The complete phenotypic spectrum associated with mutations in *TLR7*, the molecular pathogenicity of such mutations, and whether all mutations in *TLR7* cause disease in the same way is yet to be fully determined. However, together with the recent description of high-penetrant mutations in *TLR8*, and in the endosomal TLR chaperone *UNC93B1*, the discoveries summarized here are providing important new insights into the biological significance of these molecules in human immunological homeostasis (15).

## Figures and Tables

**Table 1:** Genetic and clinical characteristics of reported patients harboring *TLR7* mutations leading to enhanced TLR7 signaling

Reference (identifier)	Mutation (inheritance)	Inheritance	Sex	Age at presentation	Autoimmunity	Neurological disease
(5) (family A)	c.790T > C/Y264H	De novo	F	7 years	SLE	Relapsing hemichorea
(5) **(family B: mother)**	**C.1521T > G/F507L**	**Unknown**	**F**	**25 year**s^a^	**SLE**	**Hemiplegic “CP”** ^a^
(5) **(family B: proband)**	**C.1521T > G/F507L**	**Maternal**	**F**	**9 years**	**AQP4**^+^ **NMO**	**AQP4** ^+^ **NMO**
(5) (family C)	c.82A > G/R28G	Unknown	F	18 years	SLE	No
(6) **(AGS571: proband)**	**C.1520T > C/F507S**	**Maternal**	**F**	**4 years**	**SLE**	**Cerebral vasculitis**
(6) **(AGS571: brother)**	**C.1520T > C/F507S**	**Maternal**	**M**	**2 days**	**Not reported**	**AGS-like**
(6) **(AGS571: mother)**	**C.1520T > C/F507S**	**Unknown**	**F**	**12 years**	**Probable SLE**	**Not reported**
(6) (AGS3740)	c.1582C > A/L528I	De novo	F	1 year	Evans syndrome	AGS-like
(7)	c.800C > T/P267L	De novo	F	13 mo	Anti-NMDA encephalitis + SLE	Anti-NMDA encephalitis
(8)	c.2453G > T/G818V	De novo	F	8 mo	Evans syndrome	No
(9)	c.1517T > C/F506S	De novo (somatic)	M	15 days	SLE	ICC
(10)	c.1520G > T/F507S	De novo	F	1 mo	ITP, SLE	GDD, WMD
(11)	c.1303C > T/P435S	De novo	F	2 years	SLE	Spasticity
(12) **(proband)**^b^	**C.2519T > G/L840R**	**Maternal**	**M**	**3 years**	**SLE**	**No**
(12) **(mother**)^b^	**C.2519T > G/L840R**	**Unknown**	**F**	**No clinical data**	**No clinical data**	**No clinical data**

Bold indicates familial cases. CP, cerebral palsy; F, female; GDD, global developmental delay; ICC, intracranial calcification; ITP, immune thrombocytopenia; M, male; WMD, white matter disease.

a While SLE did not manifest until age 25 years, it is possible that the cerebral palsy diagnostic label may be incorrect and the patient’s phenotype directly linked to the mutation in *TLR7*.

b Note, a brother to the proband is reported to have presented at age 1 year with hemolytic anemia and was subsequently diagnosed with SLE at age 19 years, dying at age 29 years due to a cerebrovascular accident related to the underlying SLE. No genotyping was undertaken in this individual.
